# Quorum sensing LuxR proteins VjbR and BabR jointly regulate *Brucella abortus* survival during infection

**DOI:** 10.1128/jb.00527-24

**Published:** 2025-02-27

**Authors:** Mitchell T. Caudill, S. Tristan Stoyanof, Clayton C. Caswell

**Affiliations:** 1 Center for One Health Research, Department of Biomedical Sciences and Pathobiology, Virginia Tech, VA-MD College of Veterinary Medicine 229659 , Blacksburg, Virginia, USA; University of Illinois Chicago, Chicago, Illinois, USA

**Keywords:** *Brucella*, LuxR, quorum sensing

## Abstract

*Brucella abortus* maintains an *N*-acetyl homoserine lactone quorum sensing system that consists of two LuxR proteins, VjbR and BabR, as well as two signals, dodecanoyl (C12 AHL) and 3-oxododecanoyl (3-OXO-C12 AHL) homoserine lactone. This system regulates major virulence factors that influence the bacteria’s survival during infection. We generated the first strain that lacks both LuxR proteins and found a synergistic interaction for survival in the chronic infection C57BL/6 mouse model. Transcriptomic analyses of the *∆vjbR∆babR* double-deletion strain, as well as the cognate single-deletion strains, in a rich medium with vehicle control or supplemented with an AHL signal revealed large-scale genetic dysregulation in all conditions. Moreover, the double mutant maintained a limited response to AHL, even in the absence of the LuxR proteins. We additionally found that quorum sensing regulates the denitrification pathway but found no *in vitro* differences in the ability of quorum sensing deletion strains to clear nitric oxide stress or grow under anoxic denitrifying conditions. Finally, we confirmed that BabR autoregulates its own expression, and that VjbR mildly represses BabR expression. Altogether, these experiments help further characterize the *Brucella* quorum sensing systems and indicate that further attention should be given to the joint interactions between VjbR and BabR in controlling virulence.

**IMPORTANCE:**

*Brucella abortus* is a zoonotic bacterial pathogen that uses its quorum sensing to survive within hosts. This study further characterizes that system and indicates important future lines of inquiry. We found that both quorum sensing proteins, VjbR and BabR, coordinate to maintain survival, as well as document that both quorum sensing systems appear physiologically active.

## INTRODUCTION

*Brucella abortus* is one of the causative agents of brucellosis, a globally endemic bacterial zoonotic disease (1). *B. abortus* can infect a wide range of mammals and typically establishes persistent chronic infection within its host (2). As a primarily intracellular pathogen, *B. abortus* must respond to the numerous stressors and deprivations induced by host clearance mechanisms (3, 4). The persistent infection is maintained by a stealth strategy that relies on carefully regulated genetic expression to modify bacterial metabolism and alter host immunity through secretion of effectors (5, 6). Successful bacteria that overcome clearance attempts ultimately replicate within modified vacuoles inside macrophages and other mesenchymal cells before egressing and renewing cellular infection (7, 8).

Quorum sensing has proven to be a critical regulator of *Brucella*’s virulence program that allows persistent infection (9–11). Classic gram-negative bacterial quorum sensing systems are typically composed of one or more N-acetyl homoserine lactone (AHL) synthases, which generate the AHL quormone, as well as LuxR transcriptional regulators that responds to the signal to alter gene expression (12, 13). *Brucella*’s quorum sensing system is composed of two AHL signals, dodecanoyl AHL (C12 AHL) and 3-oxododecanoyl AHL (3-OXO-C12 AHL), along with two AHL-responsive LuxR transcriptional regulators, VjbR and BabR (11, 14). The synthetic mechanism of the AHL signals is unknown, as *Brucella* lacks homologs of LuxI or LuxM, the archetypal AHL synthases; nevertheless, the signal is present in pure culture (11, 15). *Brucella* also maintains an acylase that breaks down the AHL signal, providing signal turnover and capping the maximum concentration of the AHL signal (16). *Brucella*’s AHL production appears relatively low, at least as is achieved within *in vitro* settings, compared to other gram-negative organisms (17, 18).

Of the two LuxR proteins, VjbR is the more characterized due to its role in regulating the VirB Type IV secretion system, as well as other virulence factors (14, 19–21). ∆*vjbR* strains are typically highly attenuated during infection across *Brucella* species, and these strains have been extensively tested as potential live attenuated vaccines in a variety of animal models (22–28). BabR’s regulatory roles have been less explored, but previous work has linked it to central metabolism, as well as the production of the VirB Type IV secretion system (29–31). Despite these links, a ∆*babR* strain has only been shown to be attenuated in an immunocompromised mouse model (32).

The mechanistic roles of each of the quorum system’s components have proven difficult to isolate due to the interconnected nature of the dual signals and response regulators. We sought to further characterize the quorum sensing system of *Brucella* by creating a strain of bacteria that lacked both VjbR and BabR and using this strain in conjunction with *∆vjbR* and *∆babR* strains to better probe the precise nature of how VjbR and BabR jointly regulate transcriptional expression and, ultimately, bacterial survival. Using these strains, we also conducted RNA sequencing to determine how each component of the system altered bacterial transcription, including testing the effects of both C12 and 3-OXO-C12 AHL signals on overall transcriptional patterns. To our knowledge, this is the first assessment of the responsiveness of *Brucella* to 3-OXO-C12 AHL. Finally, we used the results of the RNA-seq to test the physiological role of VjbR and BabR signaling for regulation of the denitrification response and anaerobic growth and further tested for coregulation of BabR and VjbR through β-galactosidase assays measuring BabR and VjbR promoter activity.

## RESULTS

### The ∆vjbR∆babR mutant is synergistically attenuated compared to single ∆vjbR and ∆babR mutants in a C57BL/6 model of infection

**Fig 1:**
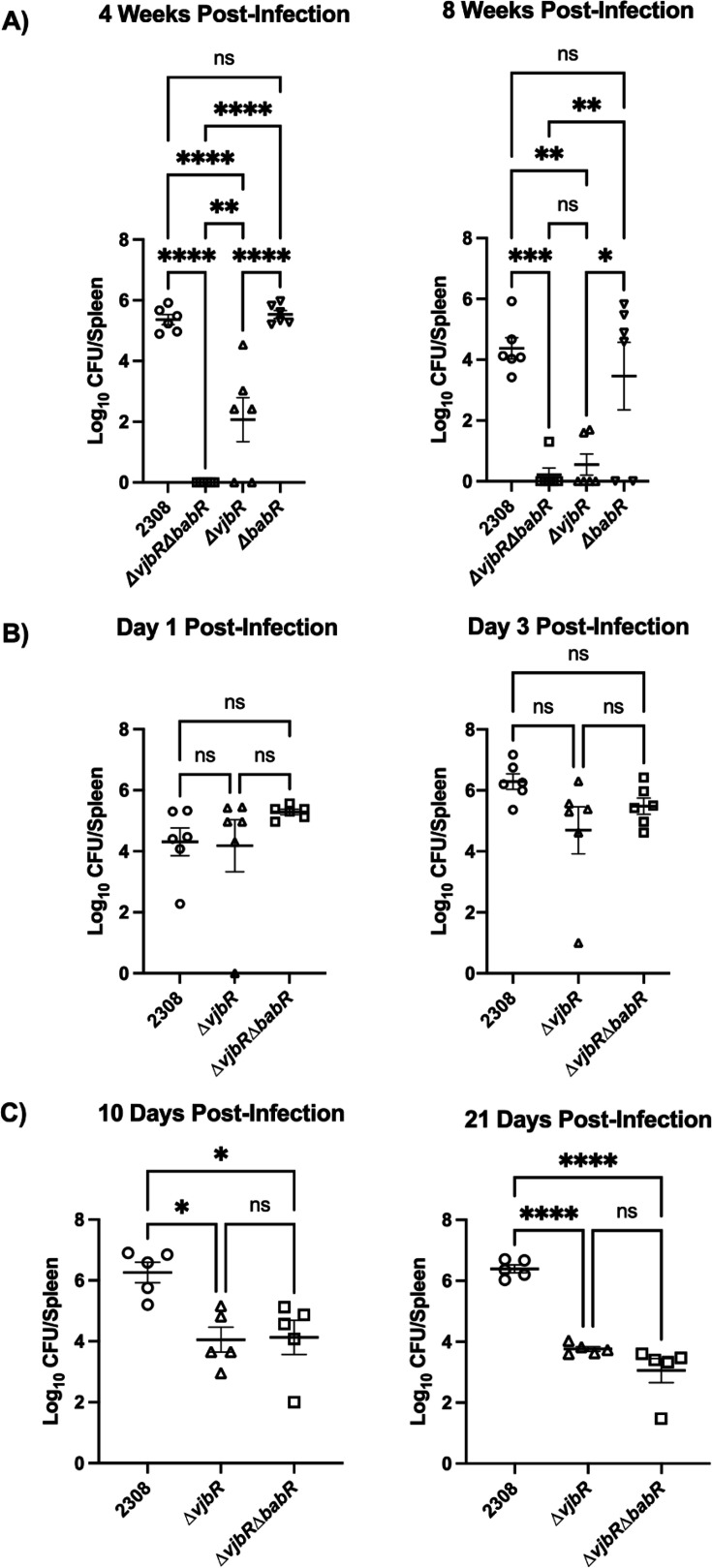
Splenic colonization of mice infected with *Brucella abortus* strains. Splenic levels of bacteria of either male and female C57BL/6 (**A**) and BALB/c male and female (**B**) and female (**C**) mice infected with the indicated strain and collected at the indicated time post-infection. Each data point represents bacteria enumerated from a single mouse spleen, with mean with standard error of the mean displayed. Statistical tests were a one-way analysis of variance with post-hoc Tukey’s test for multiple comparison. *P*-values correspond to: ns = *P* > 0.05, * = *P* ≤ 0.05, ** = *P* ≤ 0.01, *** = *P* ≤ 0.001, and **** = *P* ≤ 0.0001.

To better characterize the individual roles of VjbR and BabR during *Brucella*’s infection, we generated a ∆*vjbR*∆*babR* strain that lacked both LuxR proteins. We then tested the ability of this ∆*vjbR*∆*babR* strain to establish chronic infection in the C57BL/6 model, comparing it to wild-type infection, as well as ∆*vjbR* and ∆*babR* strains. We found that ∆*vjbR*∆*babR* was eliminated in 4 weeks by the mice, while the ∆*babR* strain showed no attenuation. The ∆*vjbR* strain was attenuated at 4 weeks, and the mice did not clear the infection until 8 weeks when again the ∆*babR* strain was not attenuated (Fig. 1A). Collectively, the difference in splenic colonization between ∆*vjbR* and ∆*vjbR*∆*babR* indicates that BabR, in the absence of VjbR, is performing functions that result in enhanced survival during infection up to the 4 week time point. Despite this, VjbR alone appears sufficient to maintain infection into the chronic state.

Given the near total clearance of the ∆*vjbR*∆*babR* strain in the C57BL/6 mice, we sought to confirm that the strain remained pathogenic and able to establish infection. We tested the ability of ∆*vjbR*∆*babR* strain to colonize the spleen of BALB/c mice, which have reduced ability to clear *Brucella* infection (Fig. 1B) (33). These results confirmed that ∆*vjbR*∆*babR* established infection in BALB/c mice while maintaining colonization at wild-type levels at 3 days post-infection. Since we could confirm the ∆*vjbR*∆*babR* could establish in the BALB/c model, and there remained the possibility that C57BL/6 mice might not allow colonization, we chose to use BALB/c mice for the remainder of our experiments. Specifically, we sought to test if we could detect differential clearance of the ∆*vjbR*∆*babR* strain compared to the ∆*vjbR* strain at either 10 or 21 days post-infection (Fig. 1C). The ∆*babR* strain was not tested given the lack of attenuation observed at 4 weeks in the C57BL/6 mice. These time points were selected to represent early inflammation prior to the typical generation of IgG for day 10 and day 21 for sustained inflammation but prior to the observed clearance in the C57BL/6 mice. At both time points, the ∆*vjbR* and ∆*vjbR*∆*babR* strains were attenuated at similar levels.

### The deletion of babR and/or vjbR, as well as addition of AHL signal, results in widespread transcriptional changes

**Fig 2:**
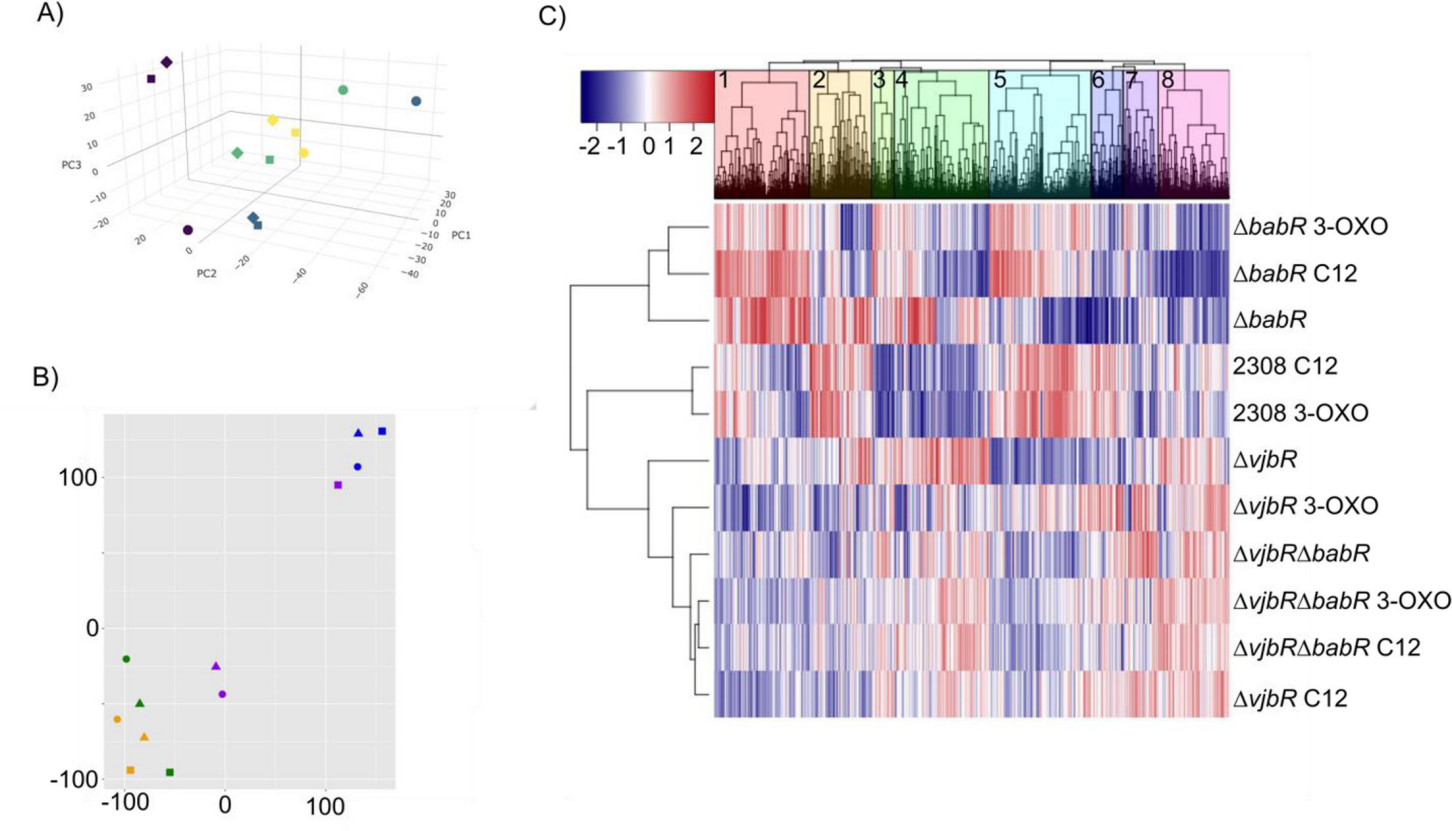
Transcriptomics reveal that the ∆*vjbR*∆*babR* strain is more similar to ∆*vjbR* than ∆*babR*, and that the presence or absence of a LuxR regulator is more impactful than the AHL signal. (**A**) Scatterplot of the principal component values 1–3 of a principal component analysis showing the average count per million reads of mRNA of strains 2308 (purple), ∆*babR* (blue), ∆*vjbR* (green), and ∆*vjbR*∆*babR* (yellow) with vehicle control (circle), C12 AHL (square), and 3-OXO AHL (diamond). A rotatable three-dimensional image is available in the Supplemental material. (**B**) *t*-Distributed stochastic neighborhood embedding plot of the same strains and conditions (3-OXO AHL is triangle). (**C**) Hierarchical clustering of the log_2_FC of strains and treatment genes relative to 2308 with vehicle control. Rows are strains with treatments, and columns are individual genes grouped into eight biological cluster numbers from left to right.

In order to better understand the transcriptional regulon of VjbR and BabR, both with and without AHL signal, we performed RNA sequencing on the ∆*babR*, ∆*vjbR*, and ∆*vjbR*∆*babR* strains along with a wild-type control. RNA was isolated from bacterial cultures grown in *Brucella* broth either supplemented with a vehicle control of dimethylformamide (DMF) or with C12 AHL or 3-OXO-C12 AHL. The vehicle control was necessary due to the poor solubility of AHL compounds in water, necessitating dissolving in an organic solvent, but proved serendipitous as discussed further below. Overall, these RNA-seq analyses reveal that the presence or absence of the LuxR regulators is more important to transcriptional state than the presence of the AHL signal, and that the presence of the AHL signal only moderately alters transcription as indicated by the close groupings of the AHL conditions to their respective vehicle control by strain. Additionally, the absence of VjbR dominates the presence of BabR as indicated by the closer groupings of ∆*vjbR*∆*babR* to ∆*vjbR* than ∆*babR* in the principal component analysis (Fig. 2A) and t-distributed stochastic neighbor embedding analysis (Fig. 2B). These relationships were also confirmed via hierarchical clustering of the transcript reads by strain (Fig. 2C).

**Fig 3:**
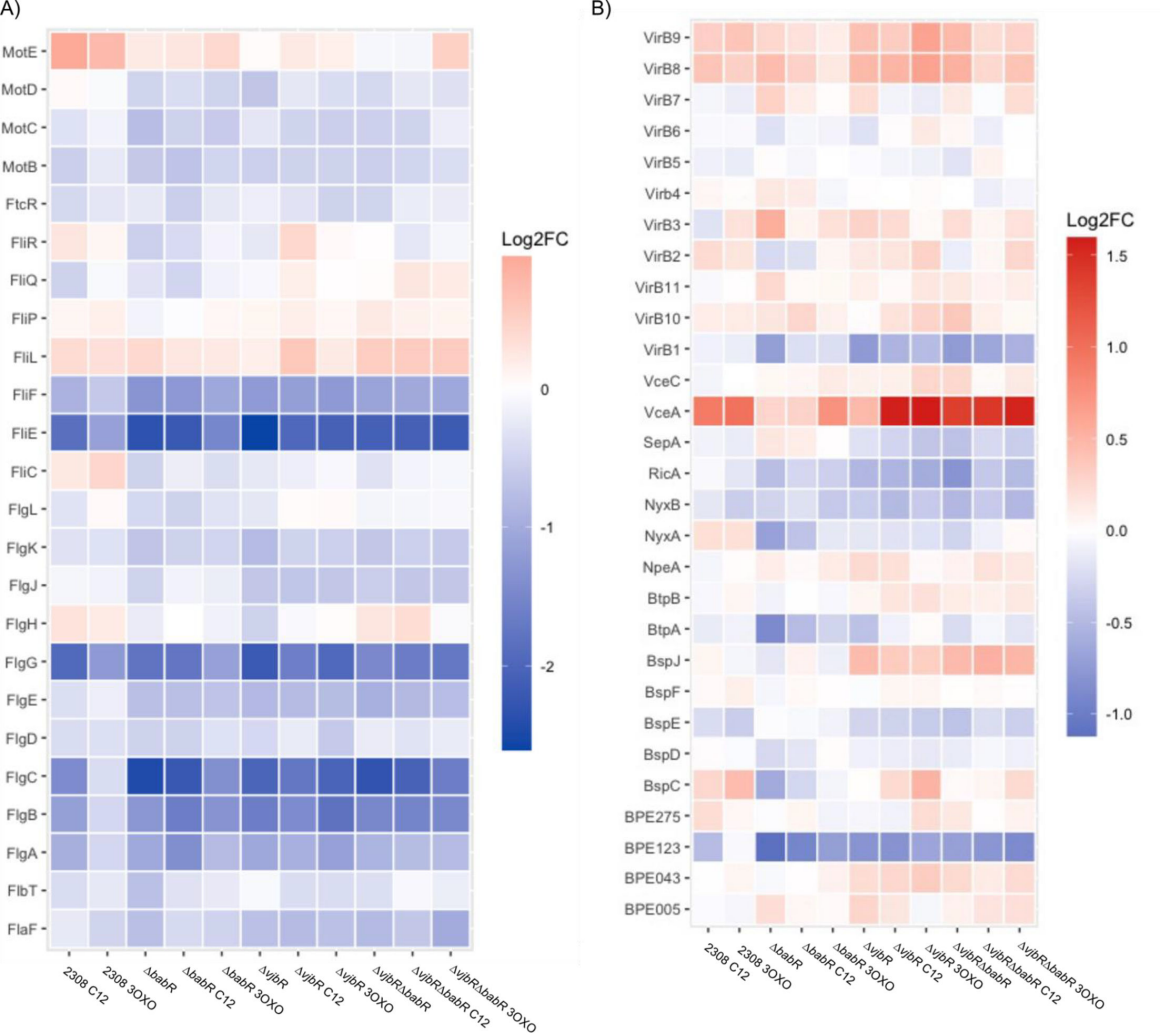
Extracellular structures and secreted effectors are diversely regulated by VjbR, BabR, and quorum signals. (**A**) Heatmap showing the relative log_2_ fold change of the listed genes relative to 2308 wild type with vehicle control for flagellar-related genes. (**B**) Heatmap showing the relative log_2_ fold change of the listed genes relative to 2308 wild type with vehicle control for Type IVB-related genes and effectors.

**Fig 4:**
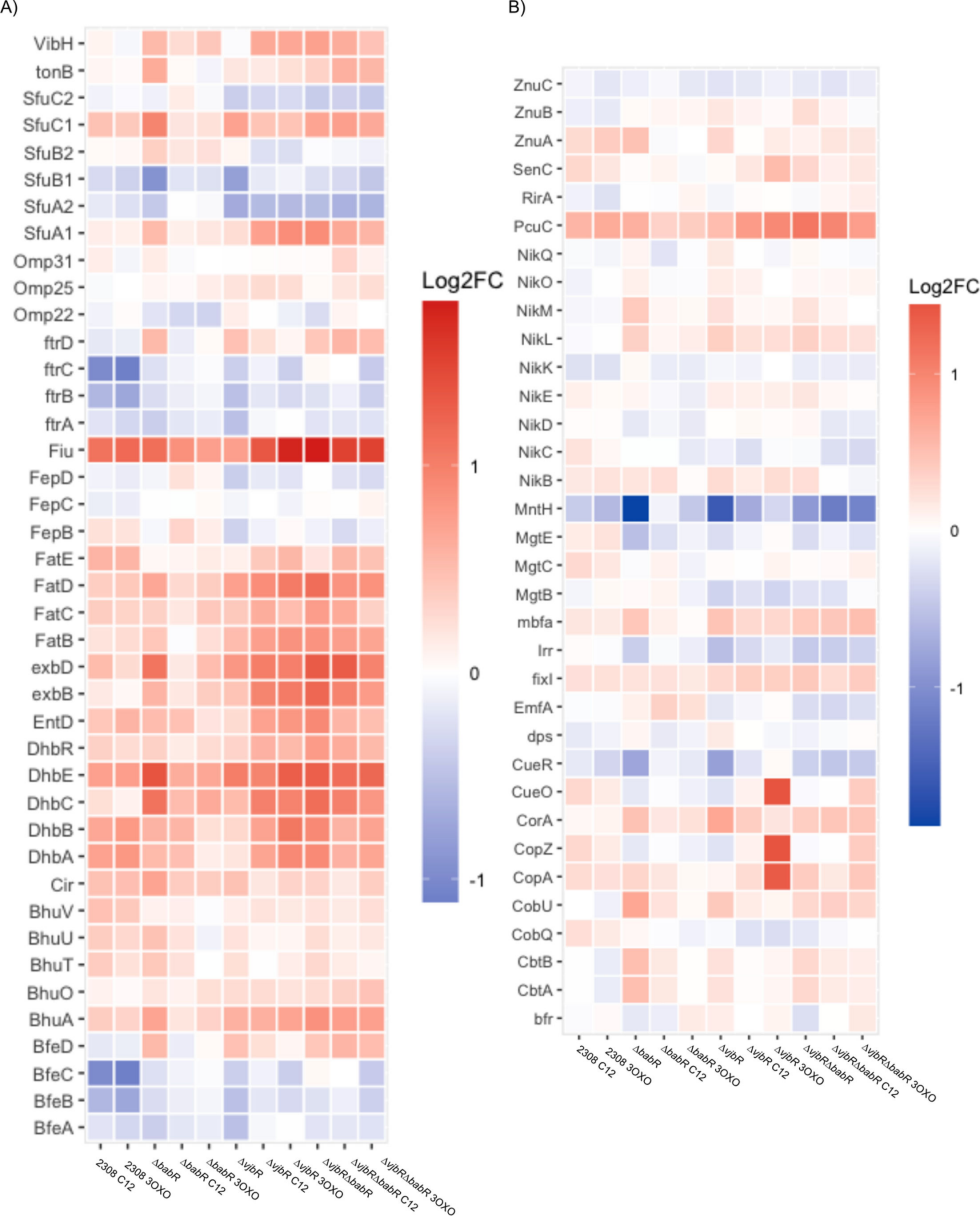
Quorum sensing suppresses heme acquisition and diversely regulates metal metabolism. (**A**) Heatmap showing the relative log_2_ fold change of the listed genes relative to 2308 wild type with vehicle control for iron related genes. (**B**) Heatmap showing the relative log_2_ fold change of the listed genes relative to 2308 wild type with vehicle control for other metal metabolism genes.

**Fig 5:**
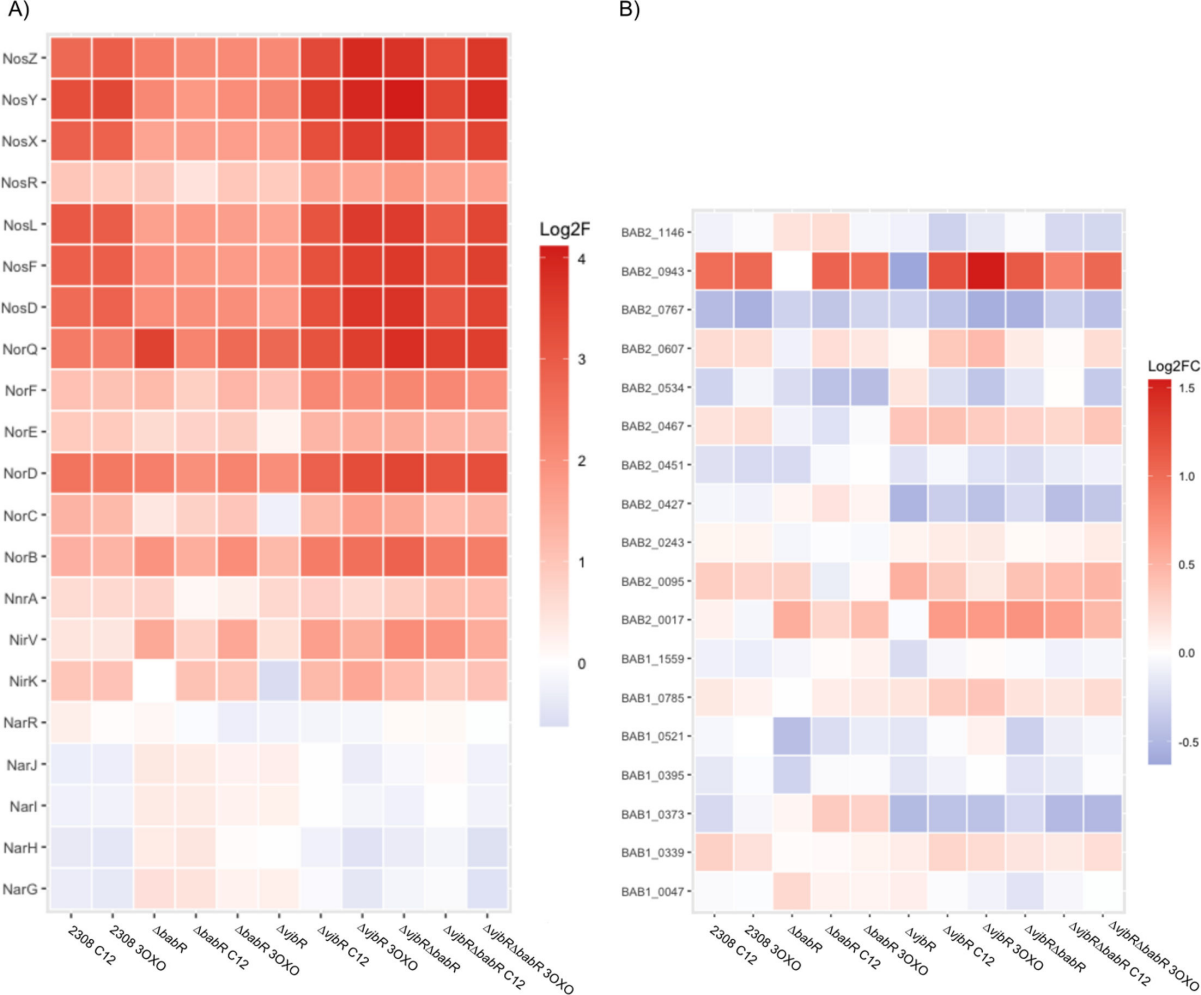
Quorum sensing represses denitrification response genes, but the regulation is not specific for twin-arginine translocation substrates. (**A**) Heatmap showing the relative log_2_ fold change of the listed genes relative to 2308 wild type with vehicle control for denitrification-related genes. (**B**) Heatmap showing the relative log_2_ fold change of the listed genes relative to 2308 wild type with vehicle control for known twin-arginine translocated gene products (35).

To further probe for potential explanations for the observed differences in the attenuation in the LuxR deletion strains, we compared transcriptional levels for subsets of genes that are of known important to *Brucella* survival and virulence. *B. abortus* produces two extracellular structures, a Type IVB secretion system used to secrete effectors, as well as a flagellum of unknown function that, nevertheless, remains important for full virulence (34). These analyses show that the quorum sensing system generally serves to activate flagellar genes while having a mixed effect on the Type IV secretion system and known effectors (Fig. 3). Interestingly, the effector VceA appears divergently regulated from VceC and repressed by both VjbR and BabR. Gene ontology analysis of the hierarchical clusters (Table S2) revealed that siderophore production was differentially regulated. To visualize this, we selected genes related to metal utilization and metabolism to compare across the variables and found that, while heme transport was repressed by the quorum system, other metal metabolism genes were diverse in their regulation by the quorum system (Fig. 4). Similarly, we observed that the denitrification system was the most highly dysregulated set of genes across the quorum sensing mutant strains (Fig. 5A). These alterations were likely potentiated by the use of DMF as a vehicle for the AHL supplements. Given that denitrification occurs in the periplasm, and critical enzymes are transported via the twin arginine translocation system, we also checked whether other known substrates of the TAT are altered, but found that the alterations caused by quorum sensing perturbations were limited to the denitrification genes (Fig. 5B) (35).

**Fig 6:**
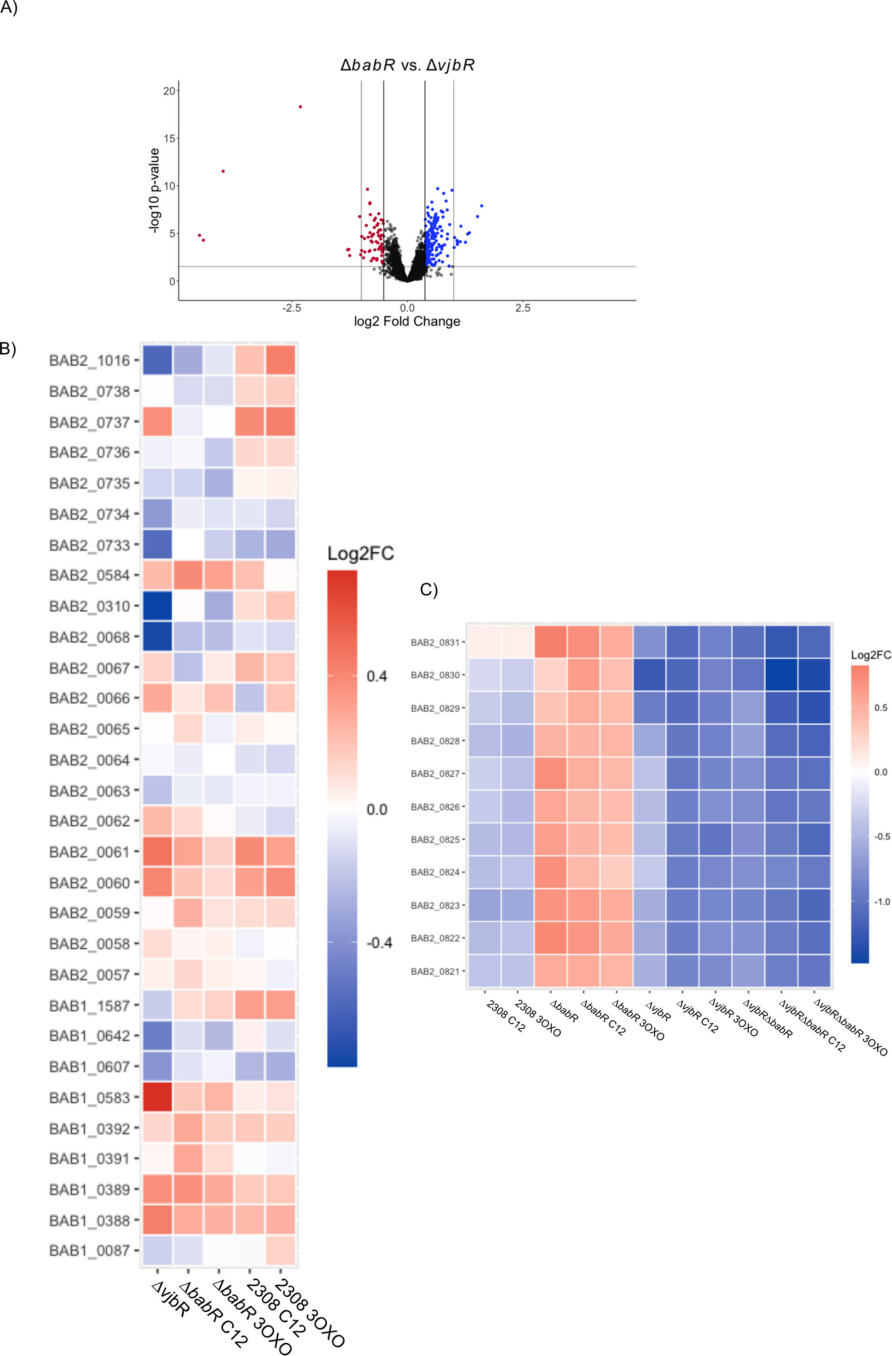
VjbR and BabR have distinct regulatory roles. The volcano plot (**A**) shows comparative genetic transcription changes of individual genes, with transcriptional directionality determined by the first strain’s level compared to the second. Potentially biologically significant genes were determined by a significant *P*-value following Benjamini–Hochberg correction for multiple comparison, indicated by the horizontal line cut off, and by a magnitude of change of at least 0.3 logs of difference represented by the inner vertical line. The outer vertical line represents a change of 2 logs. Potentially biologically significant genes with an increased level of transcription are colored blue, while those with a decreased level of transcription are colored red. The heatmap (**B**) shows a general attenuation of activity when C12 or 3-OXO C12 is present for genes known to be bound by VjbR (see Ref. [20]). The heatmap (**C**) shows a contiguous genetic area differentially regulated by BabR and VjbR that is also co-regulated by the endoribonuclease YbeY (see Discussion and Ref. [37]).

Given these large-scale changes across virulence associated genes, we sought to further characterize the specific roles of BabR and VjbR within the transcriptome data through pairwise contrast and targeted analyses. This pairwise contrast (Fig. 6A) reveals the differences in regulation between VjbR and BabR. We compared this analysis (genes available in Table S1) to the genes revealed to be VjbR and BabR, specifically by Uzureau et al., in *Brucella melitensis* (29), and found that, of the 15 genes identified as differently regulated by VjbR and BabR, only two were in our pairwise contrast (BAB1_1840 and BAB1_1355). We also sought to test whether VjbR acted as an associative or dissociative LuxR protein (see Ref. [36]) by comparing the transcriptional alterations in our data set to genes known to be directly bound by VjbR (Fig. 6B), which showed that VjbR acts as a dissociative LuxR given that the transcript level changes attenuate in the presence of C12 or 3-oxo C12 AHL. No ChIP-Seq was performed for BabR, precluding a similar analysis, but we identified an interesting ~10 kb region differentially repressed by BabR that is co-regulated by the endoribonuclease YbeY (Fig. 6C) (37).

**Fig 7:**
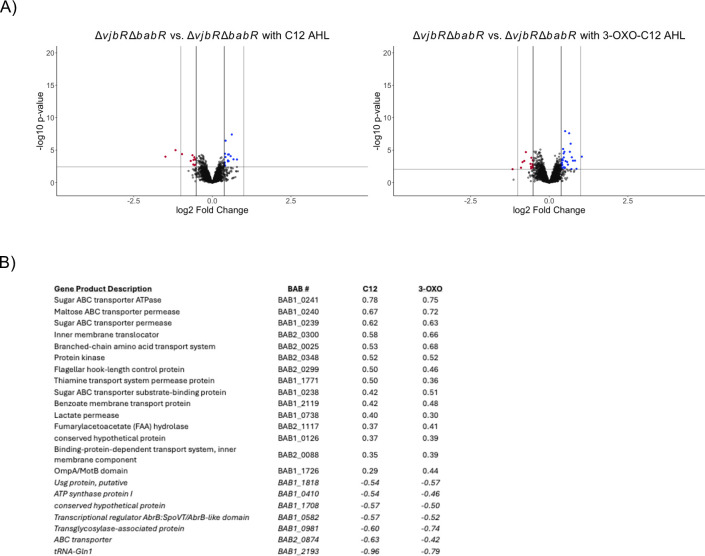
RNA-seq analysis of ∆*vjbR*∆*babR* in a rich medium with or without supplemented AHL.

Finally, and most intriguingly, we found that the ∆*vjbR*∆*babR* strain still maintained a transcriptional response to both AHL signals despite the lack of responsive LuxR proteins (Fig. 7). We believe these responses in the ∆*vjbR*∆*babR* strain are true physiological alterations rather than a random statistical fluctuation for a few reasons. First, there were statistical corrections for multiple testing with a relatively conservative false discovery rate. Second, a large proportion of the same genes are observed up-regulated in both the C12 AHL and 3-OXO-C12 AHL despite being independent cultures and tests. Finally, many genes appear to be related to sugar transport, indicating a shared physiological pathway or response (Fig. 7). Potential mechanisms for this phenomenon are elaborated in the Discussion section and colored blue, while those with a decreased level of transcription are colored red.

The volcano plot shows comparative genetic transcription changes of individual genes with transcriptional directionality determined by the first strain’s level compared to the second. Potentially biologically significant genes were determined by a significant *P*-value following Benjamini–Hochberg correction for multiple comparison indicated by the horizontal line cut-off and by a magnitude of change of at least 0.3 logs of difference represented by the inner vertical line. The outer vertical line represents a change of 2 logs. Potentially biologically significant genes with an increased level of transcription are colored blue, while those with a decreased level of transcription are colored red.

The table in Fig. 7 lists the statistically significant genes altered in the *∆vjbR∆babR* strain in both the C12 and 3-OXO-C12 conditions. The log_2_ fold change is derived from comparing the ∆*vjbR*∆*babR* strain with the vehicle control to the condition indicated in the respective column. Up-regulated genes are highlighted in blue, while down-regulated ones are highlighted in red. Statistical significance was determined via dispersion analysis, with the resulting list curated via Benjamini–Hochberg correction.

### β-Galactosidase assays reveal autoregulation by BabR

**Fig 8:**
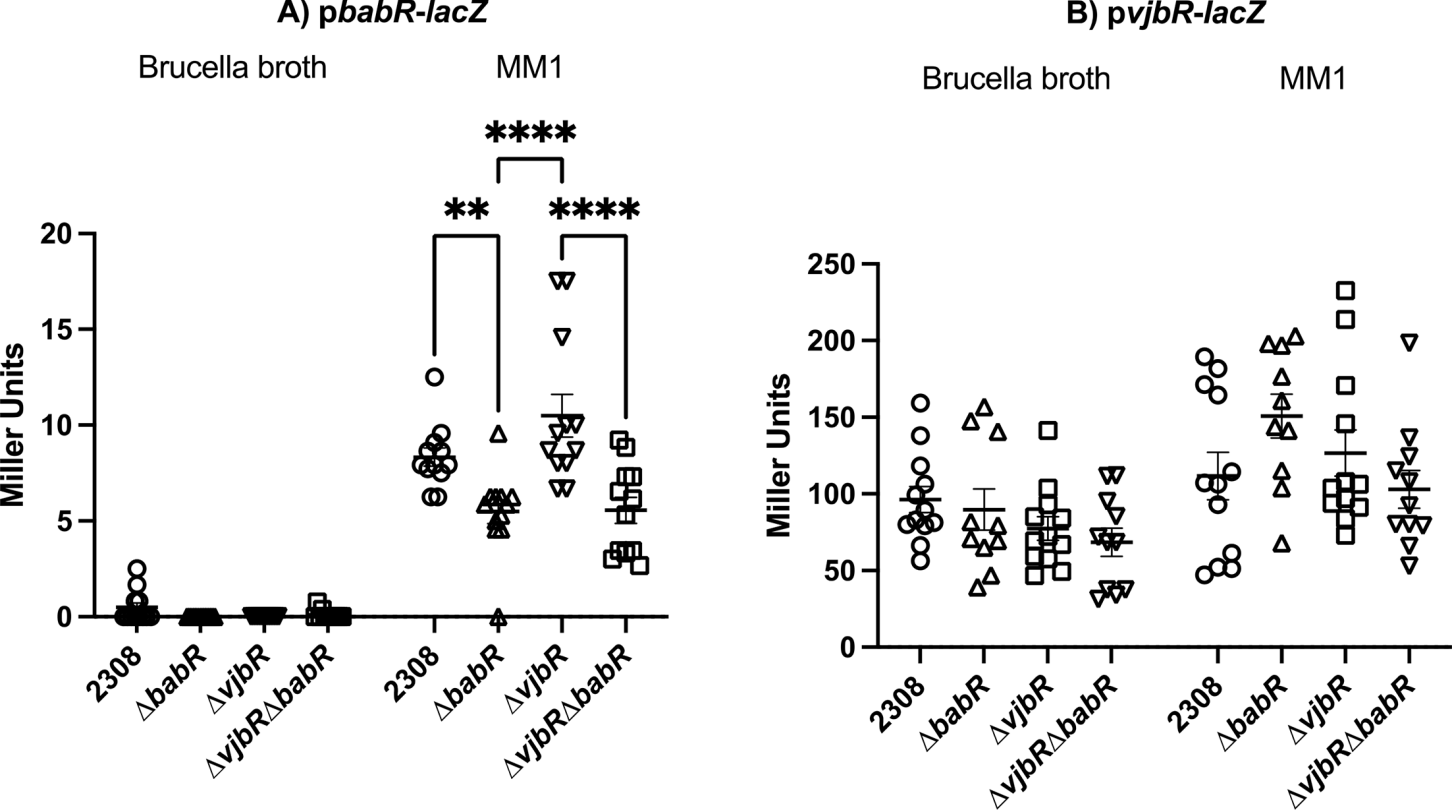
β-Galactosidase assays of BabR and VjbR promoter activity β-galactosidase assays of BabR (**A**) and VjbR (**B**) promoter activity in either brucella broth or MM1 media. Each data point represents the Miller units derived from a single culture, with the mean with standard error of the mean displayed. Statistical testing consisted of a two-way analysis of variance with post-hoc pairwise multiple comparisons. Data are aggregated from three independent experiments for each strain for both A and B. Experimentally obtained negative units were set to 0 to represent lack of promoter activity.

The RNA sequencing data showed that deletion of either VjbR and BabR altered transcription of the alternate LuxR regulator (~0.9 fold change of BabR in the ∆*vjbR* background and 1.3-fold change of VjbR in the ∆*babR* background). We sought to more specifically test the effects of VjbR and/or BabR deletion on *vjbR* and *babR* transcription, as well as the role each protein plays in its own regulation via a β-galactosidase assay. To do this, we constructed a fusion of the promoter and 5′ UTR of each gene to a plasmid containing a promoterless β-galactosidase. It has also been shown that the amount of VjbR is post-transcriptionally regulated via HutC in a urocanic acid-dependent manner (38). The design of our LacZ-fusion plasmid allowed us to additionally test whether this post-transcriptional mechanism is dependent on the VjbR promoter and 5′ untranslated region by testing for increased β-galactosidase activity in urocanic supplemented minimal media (MM1). Since we were testing VjbR, we also decided to test whether BabR levels were altered in MM1 media conditions. We found that *babR* transcript activity was significantly lowered in ∆*babR* background in MM1 media, indicating autoregulatory activation of BabR on *babR* transcription (Fig. 8). In the β-galactosidase assay in MM1, VjbR presence had no statistically significant effect on BabR promoter activity, and the presence of BabR had no effect on *vjbR* promoter activity in this assay. We additionally found that MM1 media resulted in generally increased transcription, but no physiologically meaningful differences between the strains were found.

## DISCUSSION

Throughout the study, we have sought to further characterize *Brucella*’s quorum sensing by attempting to dissect the individual roles played by the two LuxR proteins, VjbR and BabR, and their interaction with the quormone signals, C12 AHL and 3-OXO-C12 AHL. We first sought to determine the infection phenotype of the ∆*vjbR*∆*babR* strain in relation to wild type and ∆*vjbR* and ∆*babR* in C57BL/6 mice. In this mouse model, our data indicate that in the absence of VjbR, BabR maintains functions linked to bacterial survival; however, the presence of VjbR alone in the absence of BabR is sufficient to maintain infection to a chronic state (Fig. 1). Furthermore, given that the difference in ∆*vjbR*∆*babR* and ∆*vjbR* clearance did not occur until after 4 weeks in the C57BL/6 mice, there are likely shifts occurring in the host–pathogen balance in the chronic stage that are BabR-dependent or that VjbR alone cannot adequately maintain. Supporting this hypothesis is the fact that differential attenuation between ∆*vjbR* and ∆*vjbR*∆*babR* was not observed in BALB/c mice at earlier time points (Fig. 1B and C). Given that both the strain of mouse and length of infection mediate the immune clearance of the bacteria, future mechanistic studies will be needed to determine the exact role BabR plays in long-term bacterial survival. An earlier study examined *B. melitenesis* ∆*vjbR* and ∆*babR* infections in a lethal challenge of IRF-1^−/−^ C57BL/6 mice and found that, while ∆*vjbR* resulted in no mortality, ∆*babR* resulted in a mortality between that of ∆*vjbR* and 16M wild type (32). While the ∆*babR* strain had a mean CFU level at wild-type levels in our data, the differences between the clearance times of the ∆*vjbR*∆*babR* and ∆*vjbR* strains when paired with the previous findings (32) indicate that BabR contributes to wild-type virulence. Furthermore, though not statistically significant, two mice in the ∆*babR* infection group had CFUs below detectable limits, while none of the wild-type mice exhibited these undetectable levels of bacteria. Altogether, this points toward a model in which both LuxR proteins coordinate virulence during mouse infection, with VjbR playing the major role and BabR contributing a relatively minor amount.

To more fully investigate the regulons of VjbR and BabR and identify a potential mechanism for BabR’s unique contribution to long-term *Brucella* survival, we performed RNA sequencing on the ∆*babR*, ∆*vjbR*, and ∆*vjbR*∆*babR* strains in comparison to wild type. Each condition included a vehicle control (DMF) or one of the two AHL signals (C12 AHL or 3-OXO-C12 AHL). These analyses revealed extensive transcriptional changes mediated by the quorum sensing system across all strains and conditions. The presence or absence of VjbR and BabR was more impactful for the transcriptional state of the culture than the presence or absence of the AHL signal, and that the impact of VjbR deletion was epistatic to that of BabR (Fig. 2). Furthermore, despite 3-OXO-C12 AHL being at ~100-fold lower concentration than C12 AHL, 3-OXO-C12 AHL had comparable effects to C12 AHL on quorum sensing-mediated transcription, indicating that the 3-OXO-C12 generated by *Brucella* is physiologically relevant despite its minute concentration (16).

To determine how these transcriptional alterations may be linked to the attenuation phenotypes, we performed both unbiased (Fig. 2C) and targeted (Fig. 3 to 6) analyses. *Brucella* produce two extracellular structures important for virulence—a polar flagellum and a Type IVB secretion system (34). In our RNA-seq data set, the flagellar genes are activated by the presence of VjbR and BabR. A genetic circuit, in which VjbR activates flagellum expression through the transcriptional regulator FtcR, is known, but the mechanism for BabR regulation of the operon is unknown (39). Previous transcriptional activation of the flagellar genes by BabR has been reported in *B. melitensis* (32). This same study found that BabR activated Type IV secretion, which is consistent with our data on the VirB1 and VirB2 transcript levels but differs from other studies that have found that BabR represses VirB1, including our own previous work (31). The Type IV secretion system is tightly regulated, with at least multiple transcriptional regulators feeding into operon expression (40). Given this, we would suggest that the presence or absence of BabR does alter *virB1* transcript level, but that the specific effect will vary based on many metabolic factors that alter the level of all other VirB1 transcriptional regulators. The previous work referenced on BabR and *virB1* has been conducted in TSB (32), 2YT (29), low pH minimal media (31), or, in our experiments here, *Brucella* broth in a high-nitrogen stimulation. Given the diversity of results for how BabR links to *virB1* expression, it is likely that a carefully targeted study investigating isogenic strains for quorum sensing in various metabolic conditions is needed to fully resolve the link. Overall, BabR’s role in virulence remains poorly understood, and further mechanistic studies are required, particularly the regulator’s role in chronic infection.

The high-nitrogen stimulation is evidenced by the extremely high expression of denitrification genes across our data set relative to wild-type 2308 (Fig. 5A). Because AHLs are poorly soluble in water, we chose to dissolve the AHL in DMF, a nitrogen-based organic solvent. The denitrification pathway is thought to be important both for the ability of *Brucella* to survive in microaerophilic and anoxic conditions by using nitrogen as a terminal electron acceptor, as well as for surviving macrophage-generated NO stress (41–44). Our RNA sequencing analysis revealed that the denitrification pathway of *B. abortus* is regulated by the quorum sensing LuxR proteins, as evidenced by the large activation of these operons in the ∆*babR*, ∆*vjbR*, and ∆*vjbR*∆*babR* strains. Individual denitrification genes have been observed in the previous *Brucella* transcriptomic data sets of quorum sensing mutant strains, but, for our RNA sequencing data, the denitrification operons are among the most dysregulated genes, which is almost certainly driven by the choice of vehicle. Efforts to find a physiological difference among the quorum strains via *in vitro* stresses meant to induce the dysregulated operons failed to identify significant differences (see Fig. S1), but it is possible that future efforts may uncover the reason for quorum regulation of these genes.

Functionally, LuxR proteins typically activate upon binding to AHL and thereafter alter transcription through via their DNA-binding domain (15). A small number of LuxR proteins are active without AHL, with AHL signal-binding resulting in inactivation of DNA binding (36). Previous work by Weeks et al. suggested that LuxR fell into this second category based on their microarray data, which showed almost exclusive transcriptional reversal by VjbR upon the addition of C12 AHL (14). Our analyses support this finding, with genes identified in the direct VjbR regulon by Kleinman et al. via ChIP-SEQ (20), showing moderation of their transcriptional alteration upon addition of the AHL signal (Fig. 6).

We also identified a contiguous genetic region of approximately 10 kb (BAB2_0821 – BAB2_0831) that is repressed by BabR but dominantly activated by VjbR. This stretch of genes is located between known xenogeneic islands silenced by the HNS-like protein MucR and consists of two ABC transporters whose exact function is unknown, as well as a small number of enzymes (45). Intriguingly, this same stretch of genes appears within the regulon of YbeY, an endoribonuclease critical for *Brucella* survival and growth (37). The role of these ABC transporters and the reason for differential regulation by BabR and VjbR warrant further investigation as a markerless, in-frame deletion of BAB2_0822 resulting in bacterial attenuation in a macrophage model of infection (37).

A major question that remains from the data set is how the ∆*vjbR*∆*babR* strain can respond to the AHL signal despite the lack of LuxR proteins (Fig. 7). While *Brucella* possesses several other transcriptional regulators with LuxR DNA-binding domains, none have known AHL-binding domains, and most have been characterized as to other physiological roles. While it is possible that *Brucella* has a transcriptional regulator with an uncharacterized AHL-binding domain, an alternative explanation is that the AHL is sensed through altered cell membrane function. The AHL signal is lipophilic and concentrates within membranes and, at higher concentrations, can reshape the membrane structure (46–48). To our knowledge, while such reshaping has not been observed at the AHL levels utilized in our experiments, *Brucella’s* cell membrane has unique features relative to other bacteria, including an LPS with little negative charge and a highly hydrophobic cell envelope (6, 49). The general upregulation of sugar transporters and permeases suggests a stress response. It is conceivable that the ∆*vjbR*∆*babR* strain is not responding to AHL, *per se*, but rather mild membrane stress induced by the accumulation of the lipophilic AHL with vehicular DMF within its membrane. The biological significance of this finding remains to be validated.

Altogether, we have found that BabR contributes to *Brucella* virulence in the chronic state, and that BabR and VjbR jointly coordinate expression both in the presence and absence of AHL. We have found that 3-OXO-C12 induces a transcriptional response in *Brucella*, even at low nanomolar concentration, and that the quorum sensing system of *Brucella* regulates the denitrification operons. Surprisingly, we also found that a *Brucella* strain lacking LuxR proteins still transcriptionally responded to AHL. These findings advance understanding of *Brucella* biology, but the data generated also point out several new directions for better characterizing the quorum sensing system and the role it plays in *Brucella* survival and virulence.

## MATERIALS AND METHODS

### Bacteria strains and growth conditions

The strains of bacteria and plasmids used in this study are listed in Table S3. *Brucella* strains were routinely grown on Schaedler blood agar [Schaedler agar containing 5% defibrinated bovine blood (Quad Five, #910)] or in brucella broth at 37°C. For counting CFUs, *Brucella* colonies were grown on tryptic soy agar or Schaedler agar plates at 37°C. For cloning, *Escherichia coli* strains were grown on tryptic soy agar or in lysogeny broth at 37°C. When required, the medium was supplemented with appropriate antibiotics (kanamycin 45 µg/mL, carbenicillin 100 µg/mL).

### Construction of strains

Deletion strains were constructed through allelic exchange. To construct the Δ*vjbR* plasmid, approximately 1 kb lengths upstream and downstream of *vjbR* were amplified using oligonucleotides and polymerase chain reaction. The oligonucleotides were designed such that a BamH1 or HindIII restriction enzyme site was appended to the “up” and “down” arm, respectively. Following amplification, the arms were digested with the appropriate restriction enzyme. The suicide vector pNPTS138 was similarly digested, and the arms were ligated into the plasmid vector, producing a markerless deletion allele. This plasmid was confirmed via Sanger sequencing and electroporated into *B. abortus*. Merodiploid strains incorporating the plasmid were selected on Kan45 SBA plates. A selected colony from this plate was inoculated into *Brucella* broth, allowed to grow, and plated onto 10% sucrose SBA plates to allow for screening of allelic exchange via counterselection. Successful deletion was confirmed via PCR on *B. abortus* colonies using the confirmation primers listed in Table S4.

To construct the complementation plasmid, a region upstream and downstream of *vjbR* incorporating the entirety of the gene and its promoter region were amplified using oligonucleotides and polymerase chain reaction. The oligonucleotides were designed such that a *BamHI* or *PstI* restriction enzyme site was appended to the ends of the fragment. The fragment was digested with these enzymes, along with the plasmid pBBR1MCS-4. The fragment was ligated into the plasmid, and the sequence was confirmed via Sanger sequencing. The plasmid was electroporated into *B. abortus* and selected for on SBA plates containing carbenicillin..

### β-Galactosidase assays

β-Galactosidase assays were performed to measure VjbR and BabR using previously generated plasmids (31). Independent cultures of the indicated *Brucella* strains were grown to a stationary phase (typically OD_600_ of ~2) in *Brucella* broth supplemented with kanamycin at 37°C shaking to 200 rpm, then diluted to an OD_600_ of 0.5 into duplicate samples. One set of the samples had the assay performed immediately. The other samples were washed with PBS, and then resuspended in minimal media 1 supplemented with urocanic acid (50). The MM1 samples were incubated for another 4 h at 37°C shaking to allow for protein translation. For both sets of samples, the β-galactosidase activity was measured through lysing of the cells with SDS and chloroform, followed by addition of ONPG for colorimetric change. Optical density of the samples was measured at OD_420_ and OD_550_, and the Miller units were calculated using the following formula: 1,000 ◦ (Absorbance OD_420_ – (1.75 ◦ Absorbance OD_550_)) / (time of ONPG incubation ◦ volume of cells ◦ Absorbance OD_600_). For the *Brucella* broth, samples were blanked to a 9:1 mixture of Buffer Z to brucella broth, while for MM1 samples, samples were blanked to a 9:1 mixture of Buffer Z to MM1. This resulted in a situation where for low to no β-galactosidase activity, Miller units could be negative. For graphing purposes, these values were set to 0.

### Mouse virulence assays

A supplemental file describing the full methods of mouse studies in line with ARRIVE 2.0 guidelines is available in the Supplemental materials. In brief, infection of mice to determine the degree of virulence for *Brucella* strains was performed in line with previous studies (51). For Fig. 1A and B, three 6- to 7-week-old male and female C57BL/6 or BALB/c mice (Invigo order codes 044 and 162) (six mice in total per time point per strain) were injected intraperitoneally with ~100,000 bacterial CFUs of *Brucella* suspended in sterile PBS. At times indicated in the analysis, mice were humanely euthanized in order of convenience, and the spleen was aseptically removed and homogenized, with the bacterial CFUs being calculated from serial dilutions of spleen homogenate. For 1C, five female mice per strain were infected in the manner described above (JAX, #000651).

### RNA sequencing and analysis

*Brucella* strains were grown in duplicate in brucella broth at 37°C shaking at 200 to an exponential phase. The cultures were then supplemented with either DMF as a vehicle control (550 µM) or DMF containing dodecanoyl-L-homoserine lactone (Sigma, 68224) suspended to a final concentration of ~150 nM per culture or DMF containing 3-oxo- dodecanoyl-L-homoserine lactone (Sigma, O9139) suspended to a final concentration of ~1 nM per culture. Following 30 min to allow transcriptional alteration, an equal volume of 1:1 ethanol–acetone solution was added to the cultures, and the samples were stored at −80°C. RNA was isolated as previously described using a TRIzol-based extraction (31). The quality of the RNA samples was assessed using a commercial service (Genewiz Azenta), a library prepared using standard reagents, and the samples were sequenced on an Illumina NovaSeq platform. Samples consisted of two independent replicates per sample plus a pseudoreplicate consisting of equal amounts of RNA from each independent replicate. Resulting reads were aligned to the *B. abortus* genome, and differential levels of expression in genes were determined with dispersion analysis using the R-package EdgeR, with the resulting list curated via Benjamini–Hochberg correction to correct for multiple testing and determine the final significantly altered genes (52–55). In order to align with previous literature (29), genes were considered biologically significant if there was a greater than 0.3-fold change in expression.

Volcano plots were generated in R with ggplot2 (56). Principal component analysis values were calculated via prcomp in native R then graphed with plotly (57). *t*-Distributed stochastic neighborhood embedding was conducted using Rtsne (58). Hierarchical clustering was generated and plotted with native R. Heatmaps were generated via heatmap2 (59).

## Data Availability

The generated materials are available upon request pending university material transfer agreements. Sequencing data have been uploaded to NCBI under the described accession numbers PRJNA1207544. Code to generate RNA-sequencing volcano plots is available upon request.

## SUPPLEMENTAL MATERIAL

PCA-3D jb.00527-24-s0001.html 3D file for PCA.

Supplemental material jb.00527-24-s0002.docx Files S1 to S3, Fig. S1, and Table S3.

Table S1 jb.00527-24-s0003.xlsx Pairwise contrasts of differential gene expression analysis of the RNA-seq dataset

Table S2 jb.00527-24-s0004.xlsx Gene ontology analysis of hierarchical clusters identified in Figure 2C.

ASM does not own the copyrights to Supplemental Material that may be linked to, or accessed through, an article. The authors have granted ASM a non-exclusive, world-wide license to publish the Supplemental Material files. Please contact the corresponding author directly for reuse.
