# Supplementary material for: Quorum sensing LuxR proteins VjbR and BabR jointly regulate Brucella abortus survival during infection
Source: J Bacteriol. 2025 Feb 27;207(3):e00527-24. doi: 10.1128/jb.00527-24 (PMC11925318; doi:10.1128/jb.00527-24)
Supplement: Supplemental material — Files S1 to S3, Fig. S1, and Table S3. [file jb.00527-24-s0002.docx]

Overview of Supplemental Files

Supplemental File 1 – MDAR checklist

Supplemental File 2 – In Depth Methods of Mouse Studies

Supplemental File 3 – Rotatable PCA (separate html file)

Supplemental Table 1 – Pairwise contrasts of differential gene expression analysis of RNA-seq dataset (separate Excel file)

Supplemental Table 2 – Gene ontology analysis of hierarchical clusters identified in Figure 2C (separate Excel file)

Supplemental Table 3 – Primers, strains and plasmids used in study

Supplemental Figure 1 – Stress assays

**Supplemental File 1. MDAR Checklist** (Chambers, 2019)

**Materials:**

| **Newly created materials** | **indicate where provided: page no/section/legend)** | **n/a** |
| --- | --- | --- |
| The manuscript includes a dedicated "materials availability statement" providing transparent disclosure about availability of newly created materials including details on how materials can be accessed and describing any restrictions on access. | Materials and Methods |  |
|  |  |  |
| **Antibodies** | **indicate where provided: page no/section/legend)** | **n/a** |
| For commercial reagents, provide supplier name, catalogue number and [RRID](https://scicrunch.org/resources), if available. | Materials and Methods |  |
|  |  |  |
| **DNA and RNA sequences** | **indicate where provided: page no/section/legend)** | **n/a** |
| **Short novel DNA or RNA including primers, probes:** Sequences should be included or deposited in a public repository. | Deposited to NCBI Genbank |  |
|  |  |  |
| **Cell materials** | **indicate where provided: page no/section/legend** | **n/a** |
| **Cell lines:** Provide species information, strain. Provide accession number in repository **OR** supplier name, catalog number, clone number, **OR** RRID. |  | X |
| **Primary cultures:** Provide species, strain, sex of origin, genetic modification status. |  | X |
|  |  |  |
| **Experimental animals** | **indicate where provided: page no/section/legend)** | **n/a** |
| **Laboratory animals or Model organisms:** Provide species, strain, sex, age, genetic modification status. Provide accession number in repository **OR** supplier name, catalog number, clone number, **OR** RRID. | Materials and Methods |  |
| **Animal observed in or captured from the field:** Provide species, sex, and age where possible. |  |  |
|  |  |  |
| **Plants and microbes** | **indicate where provided: page no/section/legend)** | **n/a** |
| **Plants:** provide species and strain, ecotype and cultivar where relevant, unique accession number if available, and source (including location for collected wild specimens). |  | X |
| **Microbes:** provide species and strain, unique accession number if available, and source. | Materials and Methods, supplemental materials |  |
|  |  |  |
| **Human research participants** | **indicate where provided: page no/section/legend) or state if these demographics were not collected** | **n/a** |
| If collected and within the bounds of privacy constraints report on age, sex and gender or ethnicity for all study participants. |  | X |

**Design:**

| **Study protocol** | **indicate where provided: page no/section/legend)** | **n/a** |
| --- | --- | --- |
| If study protocol has been pre-registered, provide DOI. For clinical trials, provide the trial registration number **OR** cite DOI. |  | X |
|  |  |  |
| **Laboratory protocol** | **indicate where provided: page no/section/legend)** | **n/a** |
| Provide DOI **OR** other citation details if detailed step-by-step protocols are available. |  | X |
|  |  |  |
| **Experimental study design (statistics details)** | | |
| **For in vivo studies:** State whether and how the following have been done | **indicate where provided: page no/section/legend. If it could have been done, but was not, write not done** | **n/a** |
| Sample size determination | Samples sizes were determined via convivence |  |
| Randomisation | No randomization was performed |  |
| Blinding | No blinding was performed |  |
| Inclusion/exclusion criteria | A few data points excluded through due to contamination of plates or clear technical error during performance of experiments. A couple data points removed in β-galactosidase results due to incongruous result indicating likely technical error (erroneously low or high) |  |
|  |  |  |
| **Sample definition and in-laboratory replication** | **indicate where provided: page no/section/legend** | **n/a** |
| State number of times the experiment was replicated in laboratory. | Indicated in figure legends |  |
| Define whether data describe technical or biological replicates. | Indicated in Materials and Methods and figure legends |  |
|  |  |  |
| **Ethics** | **indicate where provided: page no/section/legend** | **n/a** |
| **Studies involving human participants:** State details of authority granting ethics approval (IRB or equivalent committee(s), provide reference number for approval. |  | X |
| **Studies involving experimental animals:** State details of authority granting ethics approval (IRB or equivalent committee(s), provide reference number for approval. | Materials and methods, approval number not provided |  |
| **Studies involving specimen and field samples:** State if relevant permits obtained, provide details of authority approving study; if none were required, explain why. |  | X |
|  |  |  |
| **Dual Use Research of Concern (DURC)** | **indicate where provided: page no/section/legend** | **n/a** |
| If study is subject to dual use research of concern regulations, state the authority granting approval and reference number for the regulatory approval. |  | X |

**Analysis:**

| **Attrition** | **indicate where provided: page no/section/legend** | **n/a** |
| --- | --- | --- |
| Describe whether exclusion criteria were preestablished. Report if sample or data points were omitted from analysis. If yes report if this was due to attrition or intentional exclusion and provide justification. |  | X |
|  |  |  |
| **Statistics** | **indicate where provided: page no/section/legend** | **n/a** |
| Describe statistical tests used and justify choice of tests. | Described in figure legend |  |
|  |  |  |
| **Data availability** | **indicate where provided: page no/section/legend** | **n/a** |
| For newly created and reused datasets, the manuscript includes a data availability statement that provides details for access or notes restrictions on access. | Materials and Methods |  |
| If newly created datasets are publicly available, provide accession number in repository **OR** DOI **OR** URL and licensing details where available. | Materials and Methods |  |
| If reused data is publicly available provide accession number in repository **OR** DOI **OR** URL, **OR** citation. |  | X |
|  |  |  |
| **Code availability** | **indicate where provided: page no/section/legend** | **n/a** |
| For all newly generated custom computer code/software/mathematical algorithm or re-used code essential for replicating the main findings of the study, the manuscript includes a data availability statement that provides details for access or notes restrictions. | Materials and Methods |  |
| If newly generated code is publicly available, provide accession number in repository, **OR** DOI **OR** URL and licensing details where available. State any restrictions on code availability or accessibility. |  | X |
| If reused code is publicly available provide accession number in repository **OR** DOI **OR** URL, **OR** citation. |  | X |

**Reporting**

MDAR framework recommends adoption of discipline-specific guidelines, established and endorsed through community initiatives. Journals have their own policy about requiring specific guidelines and recommendations to complement MDAR.

| **Adherence to community standards** | **indicate where provided: page no/section/legend** | **n/a** |
| --- | --- | --- |
| State if relevant guidelines (e.g., ICMJE, MIBBI, ARRIVE) have been followed, and whether a checklist (e.g., CONSORT, PRISMA, ARRIVE) is provided with the manuscript. | Materials and Methods |  |

**Supplemental File 2 – In Depth Methods of Mouse Studies**

Experiments Conducted for Figure 1A

This experiment was conducted to determine the relative virulence of three mutant bacterial strains (∆*vjbR*, ∆*babR*, ∆*vjbR*∆*babR*) compared to a wildtype control infection (2308) at 4 and 8 weeks after infection. The experimental unit was individual mice. Three 6- to 7-week old male and female C57BL/6 mice per strain per time point (24 mice total, 6 per time group per time point) were used. Sample sizes were selected based on modified use of mouse models within the field (Sola-Landa, 2002), which historically have used 5 female mice, which were then increased to 6 mice (three of each sex) to allow statistical calculations. No *a priori* criteria were set for including or excluding animals for the experiment, and no data from mice were excluded from the analysis.

Mice were allowed to acclimatize in ABSL-1 housing for approximately 36 hours after arrival with five mice of the same sex per cage, before being transferred to three mice of the same sex per cage and moved into BSL-3 conditions for infection and housing. Mice were injected intraperitoneally with ~100,000 bacterial colony forming units (CFUs) of *Brucella* suspended in sterile PBS in order of convenience with experimentalists unblinded to injection conditions and remained unblinded throughout the experiment. Mice were then cohoused by strain and sex within a single unit in an ABSL3 environment, with cages placed via convenience. During experiment, mice were provided *ad libitum* food and water, sufficient nesting material, and enrichment. No adverse effects were observed, and no humane endpoints (veterinarian recommendation following observation of lethargy, anorexia, labored breathing or ruffled coat) were exercised.

At time points indicated in the analysis, mice were humanely euthanized in order of convenience with carbon dioxide followed by cervical dislocation in accordance with IACUC approved protocols. The outside of the mice was sterilized with 70% alcohol, and the spleens were aseptically removed via sharp dissection. Spleens were homogenized and serial dilutions were plated on TSA plates and/or 1/10th of the total homogenate plated onto a TSA Kirby-Bauer plate. Colony forming units were then counted approximately three days after plating, with the number of bacterial colonies serving as the experimental outcome.

Statistical analysis was conducted using GraphPad Prism (v9.5.1). The bacterial colony counts were statistically tested via a repeated measures one-way ANOVA with post-hoc Tukey’s multiple comparisons test to determine differences between groups. Additional tests of normality were not performed. Summary statistics of the data presented in Figure 1 are reproduced below:

| 4 Weeks | | |
| --- | --- | --- |
| Strain | Mean (log10 cfu) | Standard Deviation |
| 2308 | 5.36 | 0.40 |
| ∆vjbR | 2.07 | 1.78 |
| ∆babR | 5.54 | 0.32 |
| ∆vjbR∆babR | 0 | 0 |
|  |  |  |
| 8 Weeks | | |
| 2308 | 4.38 | 0.86 |
| ∆vjbR | 0.55 | 0.85 |
| ∆babR | 3.46 | 2.72 |
| ∆vjbR∆babR | 0.22 | 0.53 |

Experiments Conducted for Figure 1B

This experiment was conducted to determine whether the ∆*vjbR*∆*babR* strain was infective at all in mice, using wildtype (2308) as a positive control, and ∆*vjbR* as a known attenuated strain that establishes infection. The experimental unit was individual mice. Three 6- to 7-week old male and female BALB/c mice per strain per time point (18 mice total, 6 per time group per time point) were used.

Sample sizes were selected based on modified use of mouse models within the field (Sola-Landa, 2002), which historically have used 5 female mice, which were then increased to 6 mice (three of each sex) to allow statistical calculations. No *a priori* criteria were set for including or excluding animals for the experiment, and no data from mice were excluded from the analysis.

Mice were allowed to acclimatize in ABSL-1 housing for approximately 60 hours after arrival with five mice of the same sex per cage, before being transferred to three mice of the same sex per cage and moved into BSL-3 conditions for infection and housing. Mice were injected intraperitoneally with ~100,000 bacterial colony forming units (CFUs) of *Brucella* suspended in sterile PBS in order of convenience with experimentalists unblinded to injection conditions and remained unblinded throughout the experiment. Mice were then cohoused by strain and sex within a single unit in an ABSL3 environment, with cages placed via convenience. During experiment, mice were provided *ad libitum* food and water, sufficient nesting material, and enrichment. No adverse effects were observed, and no humane endpoints (veterinarian recommendation following observation of lethargy, anorexia, labored breathing or ruffled coat) were exercised.

At time points indicated in the analysis, mice were humanely euthanized in order of convenience with carbon dioxide followed by cervical dislocation in accordance with IACUC approved protocols. The outside of the mice was sterilized with 70% alcohol, and the spleens were aseptically removed via sharp dissection. Spleens were homogenized and serial dilutions were plated on TSA plates and/or 1/10th of the total homogenate plated onto a TSA Kirby-Bauer plate. Colony forming units were then counted approximately three days after plating, with the number of bacterial colonies serving as the experimental outcome.

Statistical analysis was conducted using GraphPad Prism (v9.5.1). The bacterial colony counts were statistically tested via a two-way ANOVA to determine differences between groups. Additional tests of normality were not performed. Summary statistics of the data presented in Figure 1B are reproduced below:

| Day 1 | | |
| --- | --- | --- |
| Strain | Mean (log10 cfu) | Standard Deviation |
| 2308 | 4.31 | 1.12 |
| ∆vjbR | 4.19 | 2.09 |
| ∆vjbR∆babR | 5.29 | 0.21 |
|  |  |  |
| Day 3 | | |
| 2308 | 6.29 | 0.62 |
| ∆vjbR | 4.70 | 1.89 |
| ∆vjbR∆babR | 5.49 | 0.65 |

Experiments Conducted for Figure 1C

This experiment was conducted to determine the relative virulence of the ∆*vjbR* and ∆*vjbR*∆*babR* strains compared to the positive control 2308 at the 10 day and 21-day time points, as well as collect blood serum for further analysis. The experimental unit was individual mice. Five 6- to 7-week old female BALB/c mice per strain per time point (30 mice total, 5 per time group per time point) were used.

Sample sizes were selected based on modified use of mouse models within the field (Sola-Landa, 2002), which historically have used 5 female mice, and based on the significance achieved and lack of sex difference in previous studies with these strains. No *a priori* criteria were set for including or excluding animals for the experiment, and no data from mice were excluded from the analysis.

Mice were allowed to acclimatize in ABSL-1 housing for approximately 24 hours after arrival with five mice of the same sex per cage. Mice were injected intraperitoneally with ~100,000 bacterial colony forming units (CFUs) of *Brucella* suspended in sterile PBS in order of convenience with experimentalists unblinded to injection conditions and remained unblinded throughout the experiment. Mice were then cohoused by strain within a single unit in an ABSL3 environment, with cages placed via convenience. During experiment, mice were provided *ad libitum* food and water, sufficient nesting material, and enrichment. No adverse effects were observed, and no humane endpoints (veterinarian recommendation following observation of lethargy, anorexia, labored breathing or ruffled coat) were exercised.

At time points indicated in the analysis, mice were humanely euthanized in order of convenience with carbon dioxide, heart blood was immediately collected, and mice underwent cervical dislocation in accordance with IACUC approved protocols. The outside of the mice was sterilized with 70% alcohol, and the spleens were aseptically removed via sharp dissection. Spleens were homogenized and serial dilutions were plated on TSA plates and/or 1/10th of the total homogenate plated onto a TSA Kirby-Bauer plate. Colony forming units were then counted approximately three days after plating, with the number of bacterial colonies serving as the experimental outcome.

Statistical analysis was conducted using GraphPad Prism (v9.5.1). The bacterial colony counts were statistically tested via a two-way ANOVA to determine differences between groups. Additional tests of normality were not performed. Summary statistics of the data presented in Figure 1C are reproduced below:
Day 10

| Strain | Mean (log CFU) | Standard Deviation |
| --- | --- | --- |
| 2308 | 6.26 | 0.75 |
| ∆*vjbR* | 4.05 | 0.92 |
| ∆*vjbR*∆*babR* | 4.13 | 1.25 |

Day 21

| Strain | Mean (log CFU) | Standard Deviation |
| --- | --- | --- |
| 2308 | 6.39 | 0.29 |
| ∆*vjbR* | 3.76 | 0.17 |
| ∆*vjbR*∆*babR* | 3.05 | 0.89 |

**Supplemental Table 2 Methods**

Genes identified in each cluster in Figure 2C were selected and gene ontological pathway enrichment was performed with ShinyGO (v0.80) on each cluster.

Ge SX, Jung D, Yao R. ShinyGO: a graphical gene-set enrichment tool for animals and plants. Bioinformatics. 2020 Apr 15;36(8):2628-9

**Supplemental Table 3. Primers, Plasmids and Strains**

| **Oligonucleotide Primers** | |  |
| --- | --- | --- |
| **Primer Name** | **Sequence** | **Restriction site** |
| VjbR Comp Fwd | gccggatccAGAATGTGTTCCAAAGTGAAACACA | BamHI |
| VjbR Comp Rev | tataagcttCCTGAAACGCGTCTGAGCTTTTCTTTT | HindIII |
| vjbR_Up_Rev_TS3 | CATCCTTGGTGATGAAACCATGCGGG |  |
| vjbR_Up_Fwd_TS3 | GCGGATCCAAGAAAGGCGAGCTTC | BamHI |
| vjbR_Dn_For_TS3 | TGATCAACATGGTCGCGCGGA |  |
| vjbR_Dn_Rev_TS3 | GCCTGCAGCAGGAGGTGAAGGAT | PstI |
| vjbR_Con_For_TS3 | ACCTCGTCATATTTTGACCTGTTATTGTG |  |
| vjbR_Con_Rev_TS3 | TCCATCACCCTCGACATGGAA TTT |  |
| M13 Fwd | GTTTTCCCAGTCACGAC |  |
| M13 Rev | CAGGAAACAGCTATGAC |  |
|  |  |  |
| **Plasmids** |  |  |
| **Plasmid Name** | **Description** | **Reference** |
| pNPTS138 | Cloning vector; contains *sacB* gene; Kan^R^ | M. R. K. Alley, unpublished data |
| ∆*vjbR* | Truncation of vjbR with 1Kb of up and down stream flanking regions in pNPTS138 | This study |
| *babR*-*lacZ* | Fusion of promotor and 5' UTR of *babR* (BAB1_0190) to lacZ of pMR10 | Caswell, 2012 |
| *vjbR*-*lacZ* | Fusion of promotor and 5' UTR of *vjbR* (BAB2_0118) to lacZ of pMR10 | Caswell, 2012 |
| pBBR1-MCS4 | Shuttle vector; Amp^R^ | Kovach, 1995 |
| *vjbR*-*comp* | pBBR1-MCS4-*vjbR* | This study |
| *babR*-*comp* | pBBR1-MCS4-*babR* | This study |
|  |  |  |
| **Strains** |  |  |
| **Strain Name** | **Description** | **Reference** |
| 2308 | Laboratory strain representing wildtype *Brucella abortus* | Jones, 1965 |
| CC058 | In-frame, markerless deletion of *babR* (BAB1_0190) in the 2308 background | Caswell, 2012 |
| CC149 | In-frame markerless deletion of *vjbR* (BAB2_0118) in the 2308 background | This study |
| CC150 | In-frame, markerless deletion of *babR* (BAB1_0190) and *vjbR* (BAB2_0118) in the 2308 background | This study |
| MC005 | Empty vector control for pBBR-MCS4 based reporters | This study |
| MC008 | Empty vector control for pBBR-MCS4 based reporters in a ∆*vjbR*∆*babR* background | This study |
| MC012 | pBBR-MCS4-*vjbR* in a ∆*vjbR*∆*babR* background | This study |
| MC013 | pBBR-MCS4-*babR* in a ∆*vjbR*∆*babR* background | This study |
| MC021 | pMR10-*babR*-lacZ in a ∆*vjbR*∆*babR* background | This study |
| MC021 | pMR10-*vjbR*-*lacZ* in a ∆*vjbR*∆*babR* background | This study |
| MC023 | pMR10-*babR*-*lacZ* in 2308 background | This study |
| MC025 | pMR10-*babR*-*lacZ* in *∆babR* background | This study |
| MC026 | pMR10-*vjbR*-*lacZ* in a *∆babR* background | This study |
| MC027 | pMR10-*babR*-lacZ in a ∆*vjbR* background | This study |
| MC028 | pMR10-*vjbR*-*lacZ* in a ∆*vjbR* background | This study |
| MC033 | pMR10-vj*bR*-*lacZ* in 2308 background | This study |

**Supplemental Figure 1 - Stress Assays**


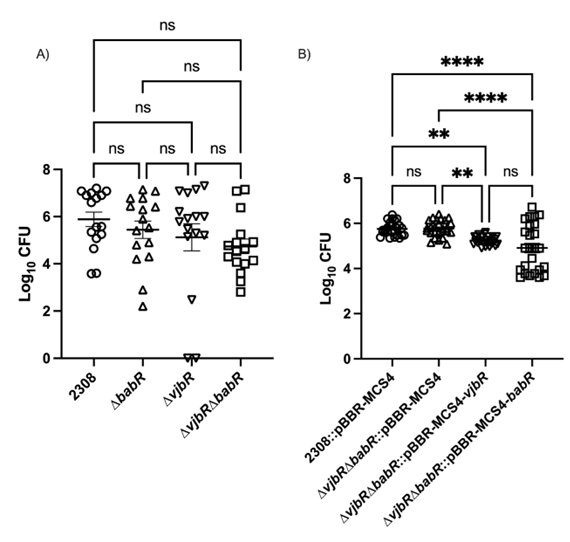


1. Growth of *Brucella* strains under nitric oxide stress

Bacteria were grown in the presence of 5 mM DETA NONOate for 18 hours at 37°C. Each data point represents the per mL CFU enumerated from the culture with the mean with standard error of the mean displayed. Statistical tests were a one-way ANOVA with post-hoc Tukey’s test for multiple comparison. P-values correspond to: ns = p > 0.05, * = p ≤ 0.05, ** = p ≤ 0.01, *** = p ≤ 0.001, **** = p ≤ 0.0001. Data is aggregated from 2 independent experiments in A, and three independent experiments in B.


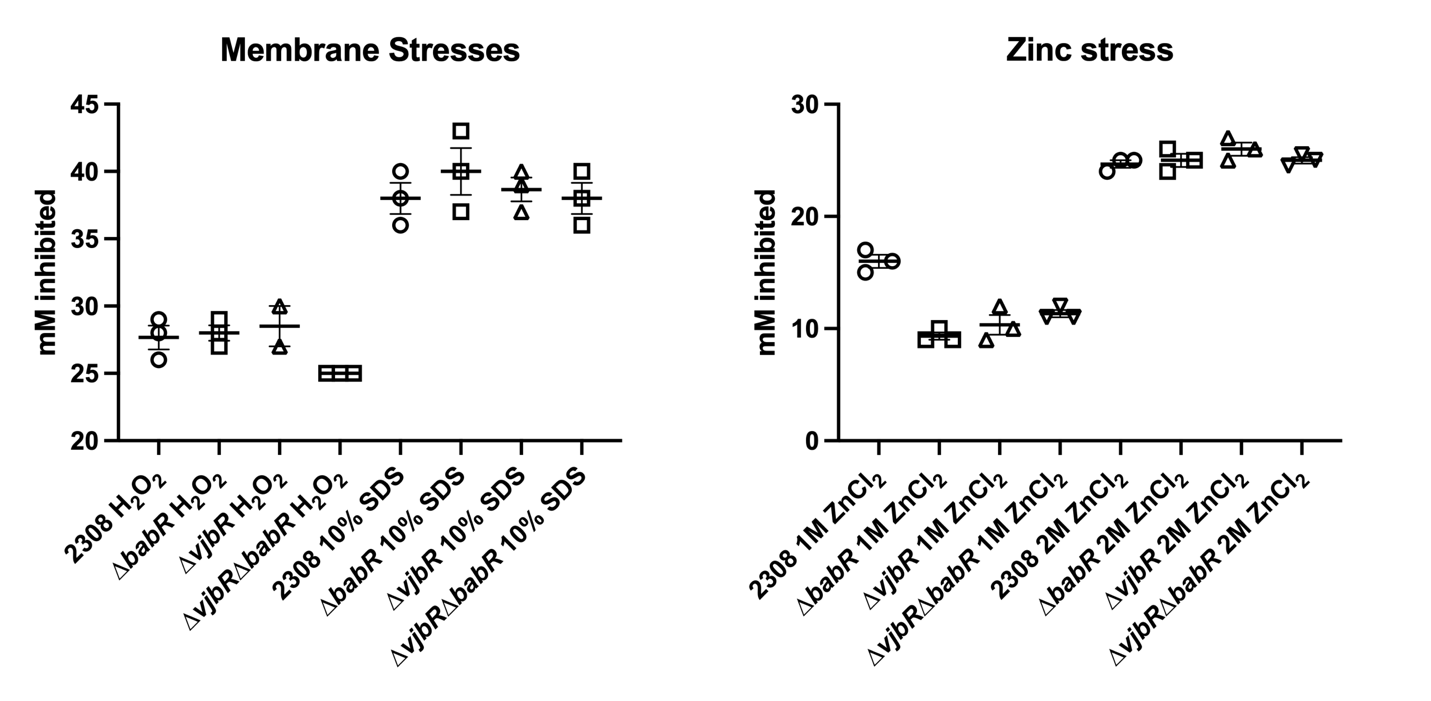


1. Disc diffusion stress assay

Bacteria were suspended at a concentration of 1x10^7^ CFU/mL in TSB with 0.6% agar and overlain on TSB agar plates. A 7 mM Whatman paper disc was placed on the center of the plate, and 7 µL of the indicated stress was infused onto the disc. Plates were incubated for 48 hrs at 37°C then the zone of inhibition was measure in millimeters. A single experiment with three replicates was performed for each stress. One data point was removed due to technical error. H_2_O_2_ stress was performed at XXX.
